# Detection of Root Physiological Parameters and Potassium and Calcium Currents in the Rhizoplane of the Apple Rootstock Superior Line 12-2 With Improved Apple Replant Disease Resistance

**DOI:** 10.3389/fpls.2021.734430

**Published:** 2021-12-17

**Authors:** Yunfei Mao, Yijun Yin, Xueli Cui, Haiyan Wang, Xiafei Su, Xin Qin, Yangbo Liu, Yanli Hu, Xiang Shen

**Affiliations:** National Key Laboratory of Crop Biology, College of Horticulture Science and Engineering, Shandong Agricultural University, Tai’an, China

**Keywords:** cultivation of resistant rootstocks, ARD, potassium and calcium currents in rhizosphere, root physiological indices, pathogen infection

## Abstract

The cultivation of resistant rootstocks is one of the more effective ways to mitigate apple replant disease (ARD). We performed an ion current test, a pot experiment, and a pathogen infection test on the apple rootstocks 12-2 (self-named), T337, and M26. The ion current test showed that exposure to ARD soil extract for 30 min had a significant effect on K^+^ ion currents at the meristem, elongation, and mature zones of the M26 rhizoplane and on Ca^2+^ currents in the meristem and elongation zones. ARD also had a significant effect on Ca^2+^ currents in the meristem, elongation, and mature zones of the T337 rhizoplane. Exposure to ARD soil extract for 5 min had a significant effect on K^+^ currents in the meristem, elongation, and mature zones of 12-2 and on the Ca^2+^ currents in the elongation and mature zones. Compared to a 5-min exposure, a 30-min exposure to ARD extract had a less pronounced effect on K^+^ and Ca^2+^ currents in the 12-2 rhizoplane. The pot experiment showed that ARD soil had no significant effect on any root architectural or physiological parameters of 12-2. By contrast, ARD soil significantly reduced some root growth indices and the dry and fresh weights of T337 and M26 compared with controls on sterilized soil. ARD also had a significant effect on root metabolic activity, root antioxidant enzyme activity (except superoxide dismutase for T337), and malondialdehyde content of T337 and M26. Pathogen infection tests showed that *Fusarium proliferatum* MR5 significantly affected the root structure and reduced the root metabolic activity of T337 and M26. It also reduced their root antioxidant enzyme activities (except catalase for T337) and significantly increased the root malondialdehyde content, reactive oxygen levels, and proline and soluble sugar contents. By contrast, MR5 had no such effects on 12-2. Based on these results, 12-2 has the potential to serve as an important ARD-resistant rootstock.

## Introduction

Apple replant disease (ARD) is a common occurrence in major apple producing areas worldwide (Mazzola and Manici, 2012; Chen et al., 2020) and has significantly limited the sustainable development of apple production (Narwal, 2010; Mao et al., 2021b). Studies have shown that ARD is caused by a complex of soil microorganisms (Mazzola and Manici, 2012; Wang et al., 2021). The ARD pathogen complex consists of oomycetes, including *Pythium* and *Phytophthora*, and fungi such as *Ilyonectria* and *Rhizoctonia*, at times acting in concert with the lesion nematode *Pratylenchus penetrans* (Zhu et al., 2016). However, the specific pathogen complex may vary across geographic regions, or even between orchards in the same region (Zhou et al., 2021). Previous studies have shown that specialized *Fusarium* spp. are the pathogenic fungi that pose the greatest threat during continuous cropping of many plants (Duan et al., 2016; Xiang et al., 2021). *Fusarium* can cause necrosis and decay of plant roots, resulting in dwarfed plants, wilting, and even tree death (Wang G. S. et al., 2018; Xiang et al., 2021). Liu (2013) and Wang G. S. et al. (2018) have shown that *Fusarium* is one of the main causes of ARD in apple orchards in the Bohai Bay region of China. Zou et al. (2014) identified *Fusarium proliferatum* and other suspected pathogenic *Fusarium* spp. from apple orchard soils in Hebei Province, China. Recently, the specialized, ARD-associated *F. proliferatum* strain MR5 (MW600437.1) has been screened, identified, and shown to be highly pathogenic to apple roots (in review).

Although there are many ways to improve ARD symptoms (Pan et al., 2017), the development of improved ARD-resistant rootstocks is a long-term effective measure to prevent and control ARD (Bowen et al., 2010). The cultivation of resistant rootstocks can effectively control pests and diseases in the replanted soil, alleviate ARD caused by certain pathogenic bacteria (Rivard et al., 2012), strengthen plant stress resistance, and increase fruit yield and quality (Zhu and Saltzgiver, 2020). There have been many studies of resistant rootstocks in Europe, the United States, and other countries. Leinfelder and Merwin (2006) reported that the growth of G30 and CG6210 plants increased significantly and steadily, and the average lifespan of the CG6210 root system was five times greater than that of M7 after 4 years. Rumberger et al. (2004) reported similar results when grafting the Royal Empire variety onto three CG rootstocks (CG16, CG30, and CG210) and two M rootstocks (M7 and M26). Rootstocks G11, G16, and G41, which were developed within the Geneva rootstock-breeding program, are reportedly tolerant to some of the causative agents implicated in ARD, although this assessment has not been confirmed consistently in all studies (Reim et al., 2019). Nonetheless, for various reasons, these rootstocks have not been promoted in China. Instead, the dwarf rootstocks T337 and M26 are still the main apple rootstocks used for production in China. T337 offers the advantages of early fruiting and large yields (Fallahi et al., 2001), and M26 has higher graft compatibility and stronger healing ability (Wang et al., 2019a). However, both these rootstocks have short lifespans and shallow root systems, and they are generally considered to be ARD “susceptible” rootstocks (Wang et al., 2019a). Therefore, it is important to specifically select and use ARD resistant rootstocks in the main apple producing areas of China.

The results of our previous research on K^+^ and Ca^2+^ absorption in the root meristem zones of three apple rootstock seedlings [*Malus hupehensis* Rehd., *Malus sieversii* (Ledeb.) Roem., and *Malus prunifolia* (Willd.) Borkh.] under replant stress showed that the greater the rootstock resistance, the less it was affected by ARD (Guo et al., 2015). K^+^ is the most abundant key cation in almost all organisms, and it plays a fundamental role in basic plant physiological processes (Adams and Shin, 2014). Ca^2+^ is also an essential nutrient for plants; it has a vital structural role in the cell wall and the maintenance of membrane integrity (Bose et al., 2011). Maintaining ion homeostasis through ion uptake and compartmentalization in the root system is crucial for normal plant growth and also for growth during stress (Gupta and Huang, 2014). In addition to the meristem zone, the elongation zone and the mature zone are also important regions of the rhizosphere. The maintenance of appropriate ion concentrations in the elongation and mature zones is important for the growth of plant roots (Sobol and Kordyum, 2009; Assaha et al., 2017; Hu et al., 2020). It is therefore important to understand whether a new rootstock with strong ARD resistance can also maintain the stability of the ion currents in each rhizosphere zone. In addition, new rootstocks should also be able to resist infection by the main harmful *Fusarium* spp. in the soil in China. *Fusarium* spp. and other soilborne fungal complexes responsible for ARD commonly cause root discoloration, root tip necrosis, and/or plant tissue necrosis at the stem base (crown rot) (Marek et al., 2013; Langenhoven et al., 2018). The root systems of highly ARD-resistant rootstocks will exhibit better performance under replant conditions (Leinfelder and Merwin, 2006).

Some crabapples (*Malus spectabilis*) are used as rootstocks for domestic apple production because they contribute beneficial characteristics (Singha, 1989). Through the patented technology of *in situ* breeding (Shen et al., 2015), our research group selected a new elite apple rootstock line named 12-2 that is tolerant to ARD. It is a new line of *M. spectabilis* that has not been identified previously. We initially selected more than 30 ARD-resistant, high-quality lines and planted them in replanted soil with 20-year-old Fuji/*Malus* × *robusta* (CarriŠre) Rehder apples in 2010. By November 2014, only 12-2 and the other superior lines survived, and the trees have continued to survive and grow vigorously to the present day (Gao, 2018; Su et al., 2021). Our previous research and aboveground measurements have shown that ARD has a significant effect on aboveground parameters of T337 and M26 but has no significant effect on 12-2 (Mao et al., 2021a). The root system is the only link connecting the aboveground parts of the plant with the soil, and root development is closely associated with plant ARD tolerance (Wang et al., 2020). Here, we further explore the potential ARD resistance of 12-2 and compare it with the *Malus* rootstocks T337 and M26, which are commonly planted in China. We specifically evaluate three aspects of rootstock performance: (1) changes in net rhizoplane K^+^ and Ca^2+^ flow in response to treatment with ARD soil extract for different durations; (2) differences in root physiological parameters between plants grown on replanted and sterilized soil; and (3) differences in tolerance toward infection by the specialized *F. proliferatum* strain MR5. Our work provides useful test materials for the breeding of resistant apple rootstocks, which are important for fundamentally solving the problem of ARD in China.

## Materials and Methods

### Experimental Sites and Materials

The ion current test, pot experiment, and pathogen infection test were conducted at the National Key Seedling Breeding Base of Shandong Agricultural University, Tai’an City, Shandong Province (36.17101°N, 117.16074°E, 134.0 masl). The ion current test and pot experiment were performed from March 2015 to October 2016. The pathogen infection test was performed from August to September 2021. The three test materials were 12-2 (self-named), a tolerant rootstock produced by our group using patented breeding technology, and T337 and M26 tissue culture rootstocks purchased from Shandong Horticultural Techniques and Services Co. Ltd. (Tai’an, Shandong, China). Beginning in early March 2015, tissue cultured seedlings of the three rootstock genotypes were subcultured under the same conditions for 8 months in modified Murashige and Skoog (MS) medium with 30 g L^–1^ sucrose, 7.5 g L^–1^ agar, 0.6 mg L^–1^ 6-BA, and 0.2 mg L^–1^ IBA, pH 5.8. Five explants were placed in each bottle of induction medium and grown in a tissue culture room at 25 ± 2°C with a 16-h light photoperiod and a light intensity of 1000 lx. In early January 2016, tissue culture explants that had been subcultured multiple times were inoculated into rooting medium (modified 1/2 MS medium with 20 g L^–1^ sucrose, 7.5 g L^–1^ agar, 0.2 mg L^–1^ 6-BA, and 1.0 mg L^–1^ IBA, pH 5.8). Five explants were placed in each bottle of induction medium and grown in a tissue culture room at 25 ± 2°C with a 16-h light photoperiod and a light intensity of 1000 lx.

### Experimental Treatments

#### Ion Current Test

On March 3, 2016, three bottles of rooted seedlings of the same size were selected from T337, M26, and 12-2. Each bottle contained two rooted tissue culture seedlings, which were used to measure rhizoplane K^+^ and Ca^2+^ ion currents with non-invasive micro-test technology (NMT) (Sun et al., 2009).

#### Pot Experiment

In early March 2016, rooted plantlets of similar size from each genotype were selected and transplanted into a sterile substrate after hardening off. At the end of March 2016, sixty plants of similar size from each rootstock genotype were selected randomly and transplanted into seedling pots (25 cm diameter, 30 cm depth) with 10 kg of soil; each pot contained three plants. Sixty plants (per genotype) were also randomly divided into two treatment combinations with ten pots (thirty plants) grown in replanted soil, and another ten pots (thirty plants) grown in sterilized soil. A total of sixty pots for the three genotypes (T337, M26, 12-2) were spaced 0.5 × 0.5 m apart at the experimental site. The replanted soil was obtained from a 20-year-old apple orchard in Xuanjiazhuang, Daolang District, Tai’an City, Shandong Province, China. The soil texture was a brown loam. The soil bulk density was 1.31 g cm^–3^, and its pH was 5.61. The soil nutrient contents included 4.56 mg kg^–1^ ammonium nitrogen, 7.38 mg kg^–1^ nitrate nitrogen, 34.82 mg kg^–1^ available phosphorus, 62.54 mg kg^–1^ available potassium, and 7.92 g kg^–1^ organic matter. The soil was passed through a 10-mesh sieve and mixed well. A portion of the replanted soil was autoclaved at 120°C for 20 min (Zealway Instrument Inc., Xiamen, Fujian, China) and used for the sterilized soil treatment. The pots were irrigated by drip irrigation every 2 days from March to May, once a day from June to September, and every 2 days in October.

#### Infection Test of Apple Replant Disease-Associated *Fusarium proliferatum* MR5

In mid-August 2021, one hundred seedlings of similar size with 4–5 leaves of T337, M26, and 12-2 were transplanted into black plastic pots (7.0 cm × 5.0 cm × 8.5 cm) filled with sterile substrate after seedling acclimatization. The specialized *F. proliferatum* strain MR5 (MW600437.1) that is associated with ARD has recently been screened and identified (in review); it is highly pathogenic to apple roots and was discovered by the research group of Professor Mao Zhiquan of Shandong Agricultural University. In early September 2021, a layer of pathogenic fungi was inoculated in liquid potato dextrose medium (PDB, Haibo, Qingdao, Shandong, China), cultured for 7 days, and then filtered through eight layers of sterile gauze to obtain a spore suspension. The concentration was measured under a microscope (Nikon Ni-U, Tokyo, Japan) using a hemocytometer (Thermo Fisher Scientific, Waltham, MA, United States), and the final concentration was adjusted to 10^6^ spores mL^–1^ with sterile water. On September 8, fifty pots of 12-2, T337, and M26 were irrigated with 20 mL spore suspension, and the other fifty pots were irrigated with an equal volume of PDB medium to serve as controls. The seedlings were grown in a tissue culture room at 24 ± 2°C with a 16-h light photoperiod and a light intensity of 1000 lx. The pots were bottom-irrigated as needed to maintain 60% soil water content.

### Measurement Indices

For each experiment (the ion current test, the pot experiment, and the pathogen infection test), there were three rootstock genotypes and a total of six treatment combinations. For each treatment combination, three bottles (two rooted tissue culture seedlings in one bottle were a biological replicate, and there were three biological replicates) containing seedlings of similar growth status were selected randomly for the ion current test. Likewise, three pots per treatment combination (three plants in one pot were a biological replicate, and there were three biological replicates) containing plants of similar growth status were selected randomly for the pot experiment, and three pots (one seedling in one pot was a biological replicate, and there were three biological replicates) of similar size and growth status were randomly selected for use in each measurement during the pathogen infection test. For all measurements, three technical replicates were performed for each biological replicate. The plants used in the ion current test were harvested on March 3, 2016. The pot experiment was harvested on October 10, 2016 after plants had grown in sterilized or replanted soil for 6 months. The measured root parameters were root architecture, root fresh and dry weights, root metabolic activity, root antioxidant enzyme activities (SOD, POD, CAT), and malondialdehyde (MDA) content. The pathogen infection test was harvested on September 13, 2021. The measured root parameters were root pathological conditions, root architecture, root fresh and dry weights, root metabolic activity, root antioxidant enzyme activities (SOD, POD, CAT), malondialdehyde (MDA) content, root reactive oxygen species levels, and root proline and soluble sugar contents.

#### Ion Currents

The roots were washed before testing, then placed in the balance solution. The solution used for the two ion measurements was the same: 0.1 mM KCl, 0.1 mM CaCl_2_, 0.1 mM MgCl_2_, 0.5 mM NaCl, 0.2 mM Na_2_SO_4_, and 0.3 mM MES. Roots were maintained in this solution for 30 min to measure the net rhizoplane ion flow before exposure to ARD (Before-ARD). The roots were then immersed in an ARD soil extract for 5 min. The extract had been prepared by mixing ARD soil with deionized water at a mass ratio of 1:1, extracting for 24 h with shaking, then filtering and storing at 4°C for later use. After equilibrating in the balance solution for 30 min, the net rhizoplane ion flow was measured after a 5-m immersion in ARD soil extract (ARD-5). Finally, the roots were immersed in ARD soil extract for 30 min. After equilibrating in the balance solution for 30 min, the net rhizoplane ion flow was measured after a 30-m immersion in ARD soil extract (ARD-30). The whole root tip was used for these measurements, and ion currents were measured 400 μm from the root tip (meristem) (Ma et al., 2018), 1000 μm from the root tip (elongation zone) (Sun et al., 2009), and 4200 μm from the root tip (mature zone) (Li and Zhang, 2014). Three root samples from each genotype were used for each measurement type (before-ARD, ARD-5, and ARD-30) and ion (Ca^2+^ or K^+^). The NMT procedure was performed as described in Sun et al. (2009) with an SIET system (BIO-001A, Younger United States, MA, United States) and MageFlux data analysis software (Xuyue Science and Technology Co., Ltd., Beijing, China).

#### Root Pathological Conditions

The pathological condition of each root system was observed with a stereomicroscope (Olympus SZX-16, Beijing, Beijing, China).

#### Root Architecture

Samples were washed with tap water and scanned with an NUScan700 scanner (MICROTEK, Shanghai, China). Total root length, surface area, volume, tip number, and branch number were analyzed with the Delta-T scan image analysis system (Delta-T Devices, Cambridge, United Kingdom).

#### Root Fresh and Dry Weights

Samples from the pot experiment and the pathogen infection test were weighted fresh and dried after root scanning. The roots and shoots of each plant were weighed separately, then placed in an oven (Suzhou DERIP Oven Manufacturing Co., Ltd., Suzhou, Jiangsu, China) at 115°C for 20 min, and finally dried at 80°C for 24 h. Weights of the dried samples were recorded.

#### Root Metabolic Activity

Root metabolic activity was measured by the 2,3,5-triphenyltetrazolium chloride (TTC) method (Ruf and Brunner, 2003). Roots were cut into approximately 1-cm pieces, placed in a test tube, and immersed in 5 mL of 0.4% TTC solution and 5 mL of phosphate buffer solution. Two milliliters of 1 M sulfuric acid solution were added to the blank tubes. Tubes were incubated in the dark at 37°C for 1 h, and 2 mL of 1 M sulfuric acid were added to stop the reaction. After standing for 20 min, the roots were gently blotted dry with absorbent paper, placed in a mortar, and ground in 3–4 mL ethyl acetate with a small amount of quartz sand. The red TTF liquid was transferred to a test tube, its volume was adjusted to 10 mL with ethyl acetate, and its absorbance was measured at 485 nm with a spectrophotometer.

#### Root Antioxidant Enzyme (Superoxide Dismutase, Peroxidase, Catalase) Activities and Malondialdehyde Content

Superoxide dismutase (SOD) activity was measured as described in Zhang et al. (2009). The amount of enzyme required to inhibit 50% of the photochemical reduction of nitroblue tetrazolium (NBT) was defined as one unit of enzyme activity, expressed as U g^–1^FW^–1^. Peroxidase (POD) activity was measured by the guaiacol method of Omran (1980) based on the change in absorbance at 470 nm. The amount of enzyme that caused an absorbance change of 0.01 per minute at 470 nm was defined as one unit of enzyme activity, expressed as U g^–1^FW^–1^ min^–1^. Catalase (CAT) activity was measured according to the method of Singh et al. (2010) based on the change in absorbance at 240 nm. The amount of enzyme that reduced the absorbance at 240 nm by 0.1 per minute was defined as one unit of enzyme activity, expressed as U g^–1^FW^–1^ min^–1^. Malondialdehyde content was measured by the thiobarbituric acid (TBA) method (Lykkesfeldt, 2001). In brief, 1 mL of supernatant was placed in a test tube, 2 mL of 0.67% TBA were added, and the mixture was heated in a boiling water bath for 15 min, then quickly placed in ice water to cool. Absorbance was measured at 600, 532, and 450 nm, and the MDA concentration was calculated as MDA (μ mol g^–1^FW^–1^) = 0.1548 (A_532_ – A_600_) – 0.01344A_450_.

#### Root Reactive Oxygen Species Levels

Root H_2_O_2_ content and O_2_^–^ production rate were measured using the methods of Bai et al. (2009). The H_2_O_2_ content was measured by ultraviolet spectrophotometry. Fresh tissue samples (2 g) were combined with 4°C pre-cooled acetone and a small amount of quartz sand at a ratio of 1:1 between the material and the solvent, ground into a homogenate, and centrifuged at 300 rpm for 10 min. A 1-mL sample of supernatant was combined with 0.1 mL 5% titanium sulfate and 0.2 mL concentrated ammonia water, then centrifuged at 5000 rpm for 10 min. The pellet was washed 3–5 times with acetone until the plant pigments had been removed; then 5 mL of 2 M sulfuric acid was added to the precipitate. When the precipitate was completely dissolved, the absorbance of the titanium peroxide complex was measured at 415 nm and compared to a standard curve to determine the H_2_O_2_ content.

O_2_^–^ production was measured by the hydroxylamine reaction. Fresh tissue samples (2 g) were combined with 1 mL 0.05 M phosphate buffer (pH 7.8) and ground in an ice bath. The mixture was centrifuged at 12000 rpm for 15 min, and 0.5 mL phosphate buffer and 1 mL 10 M hydroxylamine hydrochloride were added to 0.5 mL of the supernatant. The mixture was allowed to stand at 25°C for 1 h. Then 1 mL 17 mM P-sulfanilic acid and 1 mL 7 mM α-zeamine were added, and the mixture was allowed to stand for 20 min at 25°C. Its absorbance was measured at 530 nm and compared to a standard curve to determine O_2_^–^ production rate.

#### Root Proline and Soluble Sugar Contents

The proline content was determined by the ninhydrin colorometric method (Wang et al., 2019b). Each fresh tissue sample (0.5 g) was placed into a large test tube, combined with 5 mL 3% sulfosalicylic acid, and placed into a boiling water bath. The tube was removed from the water bath after 10 min (having been stirred evenly during the process) and filtered into a clean test tube after cooling. A 2-mL sample of the extract was placed into another clean test tube with a stopper, and 2 mL glabraic acid and 2 mL acid ninhydrin reagent were added. The mixture was heated in a water bath for 30 min and became red. After cooling, 4 mL toluene was added, and the mixture was shaken for 30 s. After standing, the upper layer was removed and placed into a 10-mL centrifuge tube, then centrifuged at 3000 rpm for 5 min. The upper toluene solution containing proline that had turned red was pipetted into a cuvette, its absorbance was measured at 520 nm with an ultraviolet spectrophotometer, and its proline content was determined by comparison with a standard curve. Toluene was used as the blank control.

The soluble sugar content was determined by the phenol-sulfuric acid method (Masuko et al., 2005). A 0.2-g sample of fresh tissue was chopped and mixed, divided among three graduated test tubes to create three technical replicates, combined with 5 mL of distilled water, sealed with plastic film, and extracted in boiling water for 30 min. The resulting extract was filtered into a 25-mL volumetric flask and brought up to a constant volume (i.e., to the flask marking). A 0.5-mL sample of the liquid was pipetted into a test tube and combined sequentially with 1.5 mL distilled water, 1 mL 9% phenol solution, and 5 mL concentrated sulfuric acid. The sample was mixed to spread the color evenly, its absorbance was measured at 480 nm, and its soluble sugar content was determined by comparison to a standard curve.

### Statistical Analyses

The data were analyzed using SPSS (version 17, IBM SPSS, Chicago, IL, United States). One biological replicate value for each treatment combination was the average of two rooted tissue culture seedlings in one bottle for the ion current test, three plants in one pot for the pot experiment, and one seedling in one pot for the pathogen infection test. Unless otherwise noted, the significance of differences among treatment means was assessed by Student’s *t*-test and Duncan’s multiple range test (DMRT) at a 0.05 probability level.

## Results and Analysis

### Rhizoplane Ion Currents

#### Rhizoplane K^+^ Ion Currents

##### Meristem Zone

In the Before-ARD samples, the root meristem zones (400 μm from the root tip) of T337, M26, and 12-2 all absorbed K^+^ from the rhizoplane (Table 1 and Supplementary Figure S1). When roots were exposed to ARD soil extract for 5 min (ARD-5), the average net K^+^ fluxes from the rhizoplane into T337 and M26 root meristems were significantly reduced, and 12-2 meristems changed from net K^+^ absorption to net K^+^ release. The average net K^+^ flux into T337 meristems increased significantly in the ARD-30 treatment relative to the ARD-5 treatment, whereas the average net K^+^ flux into M26 meristems decreased significantly. The 12-2 roots changed from releasing K^+^ in the ARD-5 treatment to absorbing K^+^ in the ARD-30 treatment. The average net K^+^ flux into 12-2 meristems was significantly higher in the ARD-30 treatment than in the Before-ARD treatment.

**TABLE 1:** Effects of ARD on net K^+^ flux (pmol cm^–2^s^–1^) of three rootstocks in the meristem zone (400 μm from the root tip).

Treatment	T337	M26	12-2
Before-ARD	–74.93 ± 2.17c	–144.38 ± 0.82c	–82.19 ± 3.33b
ARD-5	–46.28 ± 2.76a	–95.75 ± 1.06b	59.70 ± 6.24a
ARD-30	–65.59 ± 1.52b	–36.33 ± 1.49a	–84.63 ± 9.22c

*Before-ARD, net rhizoplane ion flow before ARD treatment; ARD-5, net rhizoplane ion flow after immersion in ARD soil extract for 5 min; ARD-30, net rhizoplane ion flow after immersion in ARD soil extract for 30 min. A negative flow rate indicates that the measured cations are flowing into the root, i.e., the roots are absorbing the ions. Different letters show significant differences (P < 0.05). The same conventions are used in Tables 2–6, below.*

##### Elongation Zone

Before exposure to ARD soil extract, the root elongation zones of all three rootstocks absorbed K^+^, consistent with the results from the meristem zones (Table 2 and Supplementary Figure S2). The net K^+^ flux in the T337 elongation zone did not differ significantly between Before-ARD and ARD-5, whereas the net K^+^ flux in the M26 elongation zone was significantly lower in ARD-5, and that of 12-2 changed from absorption to release. The net K^+^ flux in T337 elongation zones did not differ significantly between ARD-5 and ARD-30, whereas the K^+^ flux of M26 changed from absorption to release. The K^+^ flux of 12-2 changed back from release to absorption in ARD-30 and was significantly higher than in Before-ARD.

**TABLE 2:** Effects of ARD on net K^+^ flux (pmol cm^–2^s^–1^) of three rootstocks in the elongation zone (1000 μm from the tip).

Treatment	T337	M26	12-2
Before-ARD	–47.96 ± 1.25b	–103.26 ± 1.05c	–15.80 ± 2.55b
ARD-5	–45.74 ± 1.87ab	–74.43 ± 1.08b	126.11 ± 8.22a
ARD-30	–42.84 ± 1.53a	5.99 ± 4.19a	–52.84 ± 9.23c

##### Mature Zone

K^+^ flux data (Table 3 and Supplementary Figure S3) showed that the mature zones of T337 and M26 absorbed K^+^ before and after exposure to ARD soil extract, whereas 12-2 mature zones released K^+^. The net K^+^ flux into T337 mature root zones was significantly higher in ARD-5 than in Before-ARD; the net K^+^ flux into M26 mature root zones was significantly lower; and the net K^+^ flux out of 12-2 mature root zones was significantly higher. In the ARD-30 treatment relative to ARD-5, the net K^+^ fluxes into T337 and M26 and the net K^+^ flux out of 12-2 were significantly reduced.

**TABLE 3:** Effects of ARD on net K^+^ flux (pmol cm^–2^s^–1^) of three rootstocks in the mature zone (4200 μm from the tip).

Treatment	T337	M26	12-2
Before-ARD	–38.74 ± 1.43b	–83.93 ± 1.21c	23.92 ± 4.91c
ARD-5	–54.74 ± 3.99c	–47.02 ± 1.35b	99.42 ± 7.69a
ARD-30	–33.71 ± 4.21a	–20.77 ± 1.55a	57.08 ± 8.04b

#### Rhizoplane Ca^2+^ Ion Currents

##### Meristem Zone

Before and after exposure to ARD soil extract, the meristems of all three rootstocks showed net Ca^2+^ release (Table 4 and Supplementary Figure S4). After exposure to ARD soil extract for 5 min, the net Ca^2+^ efflux of T337 and 12-2 showed little change, and the net Ca^2+^ flux from M26 was significantly increased. After 30 min of ARD soil extract exposure, the net efflux of Ca^2+^ from T337 and M26 meristem zones was significantly increased. By contrast, net Ca^2+^ efflux from the 12-2 meristem zone was basically unchanged after 30 min of exposure.

**TABLE 4:** Effects of ARD on net Ca^2+^ flux (pmol cm^–2^s^–1^) of three rootstocks in the meristem zone (400 μm from the tip).

Treatment	T337	M26	12-2
Before-ARD	53.81 ± 3.26b	28.75 ± 2.58c	68.18 ± 3.15b
ARD-5	52.87 ± 2.42b	37.25 ± 4.14b	71.16 ± 2.29b
ARD-30	109.18 ± 4.35a	195.03 ± 7.65a	79.86 ± 3.12a

##### Elongation Zone

Before exposure to ARD soil extract, the elongation zone of T337 took up Ca^2+^ from the rhizoplane; by contrast, the elongation zones of M26 and 12-2 showed net Ca^2+^release (Table 5 and Supplementary Figure S5). After exposure to ARD soil extract for 5 min, T337 elongation zones switched from net Ca^2+^ influx to net Ca^2+^ efflux, and the Ca^2+^ efflux rates of M26 and 12-2 were significantly higher in ARD-5 than in Before-ARD. After 30 min of ARD soil extract exposure, Ca^2+^ efflux from T337 and M26 elongation zones was significantly enhanced relative to ARD-5, and this enhancement was greater in M26. By contrast, Ca^2+^ efflux of 12-2 was significantly lower in ARD-30 than in ARD-5 or Before-ARD.

**TABLE 5:** Effects of ARD on net Ca^2+^ flux (pmol cm^–2^s^–1^) of three rootstocks in the elongation zone (1000 μm from the tip).

Treatment	T337	M26	12-2
Before-ARD	–47.37 ± 3.08c	37.30 ± 5.27c	44.79 ± 2.83b
ARD-5	55.74 ± 3.32b	86.17 ± 4.15b	75.05 ± 4.72a
ARD-30	60.78 ± 3.07a	108.30 ± 5.09a	20.90 ± 3.16c

##### Mature Zone

Before and after exposure to ARD soil extract, the meristems of all three rootstocks showed net Ca^2+^ release (Table 6 and Supplementary Figure S6). After exposure to ARD soil extract for 5 min, the net Ca^2+^ fluxes of T337 and M26 mature zones were significantly lower, and this decrease was greater in M26 than in T337. The net Ca^2+^ flux of 12-2 was significantly higher after 5 min of exposure. The net Ca^2+^ efflux of T337 and M26 mature zones was greater after 30 min of exposure than after 5 min of exposure, whereas that of 12-2 mature zones was significantly lower. Compared with Before-ARD, the average Ca^2+^ efflux of T337 was significantly higher in ARD-30, whereas the average Ca^2+^ efflux of M26 and 12-2 did not differ significantly between Before-ARD and ARD-30.

**TABLE 6:** Effects of ARD on net Ca^2+^ flux (pmol cm^–2^s^–1^) of three rootstocks in the mature zone (4200 μm from the tip).

Treatment	T337	M26	12-2
Before-ARD	45.06 ± 4.15b	138.83 ± 8.01a	34.17 ± 2.05b
ARD-5	35.99 ± 6.73c	46.12 ± 4.96b	44.07 ± 3.37a
ARD-30	98.70 ± 2.52a	136.50 ± 5.27a	32.83 ± 2.86b

### Pot Experiment

#### Root Architecture

Growth on ARD soil influenced the root architecture of T337 and M26 (Table 7): ARD soil significantly reduced root system length, surface area, volume, tip number, and bifurcation number in T337 and significantly reduced the root surface area and volume of M26. ARD soil had no significant effect on root architectural parameters of 12-2.

**TABLE 7:** Effects of ARD soil on the root characteristics of 12-2, T337, and M26 apple rootstocks.

Treatment		Root length (cm)	Root area (cm^2^)	Root volume (cm^3^)	Root tips number	Root forks number	Root diameter (mm)
T337	Replanted soil	2531.22 ± 186.18	655.15 ± 52.83	13.50 ± 1.23	6553.33 ± 467.32	14380.33 ± 1324.18	0.82 ± 0.01
	Sterilized soil	4363.10 ± 18.51*	1225.43 ± 67.45*	27.61 ± 3.41*	12297.00 ± 937.54*	27376.33 ± 1754.65*	0.96 ± 0.07
M26	Replanted soil	2865.20 ± 345.76	688.14 ± 45.73	13.26 ± 0.15	8170.00 ± 963.28	14683.00 ± 1469.35	0.80 ± 0.07
	Sterilized soil	3462.33 ± 337.52	1031.53 ± 53.17*	24.58 ± 0.53*	8771.00 ± 515.36	20465.33 ± 2263.27	0.89 ± 0.08
12-2	Replanted soil	6006.08 ± 728.38	2132.55 ± 42.17	64.09 ± 8.71	18216.00 ± 1875.35	41602.00 ± 4548.34	1.08 ± 0.14
	Sterilized soil	6767.66 ± 538.23	2234.26 ± 127.68	60.12 ± 3.24	20664.33 ± 2684.36	45094.00 ± 4835.11	1.07 ± 0.03

Within a rootstock, Student’s t-test was used to determine the significance of the difference between control and ARD soil. Data are expressed as mean ± SE. *p < 0.05; **p < 0.01.

#### Root Fresh and Dry Weights

Growth on ARD soil significantly affected the above- and belowground fresh and dry weights of T337 and M26, and it had a significant effect on the root-to-shoot ratio of T337 (Table 8). There were no significant effects of ARD soil on fresh or dry weights of 12-2.

**TABLE 8:** Effects of ARD on the fresh and dry weights of 12-2, T337, and M26 apple rootstocks.

Treatment		Fresh weight (g)		Dry weight (g)		R/S
		Root	Branch	Root	Branch	
T337	Replanted soil	8.09 ± 1.38	14.14 ± 2.18	2.80 ± 0.56	7.39 ± 1.04	0.38 ± 0.03
	Sterilized soil	20.52 ± 0.63*	20.62 ± 1.68*	6.77 ± 0.22**	10.43 ± 0.61*	0.65 ± 0.02**
M26	Replanted soil	14.52 ± 2.27	10.01 ± 1.32	5.53 ± 0.87	5.24 ± 0.77	0.99 ± 0.06
	Sterilized soil	30.13 ± 1.31*	22.99 ± 0.66*	12.32 ± 0.49*	12.41 ± 0.42**	1.01 ± 0.01
12-2	Replanted soil	43.51 ± 2.74	19.66 ± 1.78	15.50 ± 1.32	9.74 ± 0.68	1.60 ± 0.13
	Sterilized soil	46.95 ± 3.61	22.25 ± 1.53	16.82 ± 1.39	11.37 ± 0.82	1.48 ± 0.02

Within a rootstock, Student’s t-test was used to determine the significance of the difference between control and ARD soil. Data are expressed as mean ± SE. *p < 0.05; **p < 0.01. R/S indicates the root-to-shoot ratio. It is the value obtained by dividing the root dry weight by the shoot dry weight.

#### Root Metabolic Activity Measurements

**FIGURE 1:**
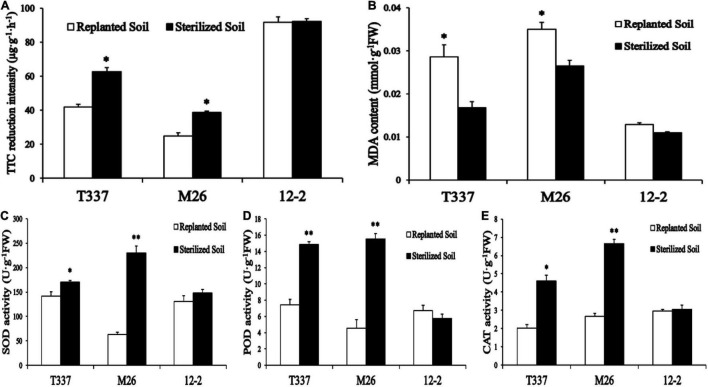
Effects of ARD soil on multiple root parameters of 12-2, T337, and M26 apple rootstocks. **(A)** Root metabolic activity was measured by the 2,3,5-triphenyltetrazolium chloride (TTC) method; **(B)** Root malondialdehyde (MDA) content; **(C)** Root superoxide dismutase (SOD) activity; **(D)** Root peroxidase (POD) activity; **(E)** Root catalase (CAT) activity. Within a rootstock, Student’s *t*-test was used to determine the significance of the difference between control and ARD soil. *p < 0.05; ^**^p < 0.01.

Growth on ARD soil had a significant effect on the root metabolic activity of T337 and M26 (Figure 1A) but not on the root metabolic activity of 12-2. The root metabolic activity of 12-2 in replanted soil was also higher than that of T337 and M26.

#### Root Antioxidant Enzyme Activity and Malondialdehyde Content

Growth on ARD soil altered the antioxidant enzyme activity and MDA content of T337 and M26 roots (Figures 1B–E). ARD soil significantly reduced POD and CAT activities in T337 and the antioxidant enzyme activity of M26; it significantly increased the MDA contents of T337 and M26. ARD soil had no significant effect on the antioxidant enzyme activity or MDA content of 12-2.

### Infection Test With Apple Replant Disease-Associated *Fusarium proliferatum* MR5

#### Root Pathological Conditions

**FIGURE 2:**
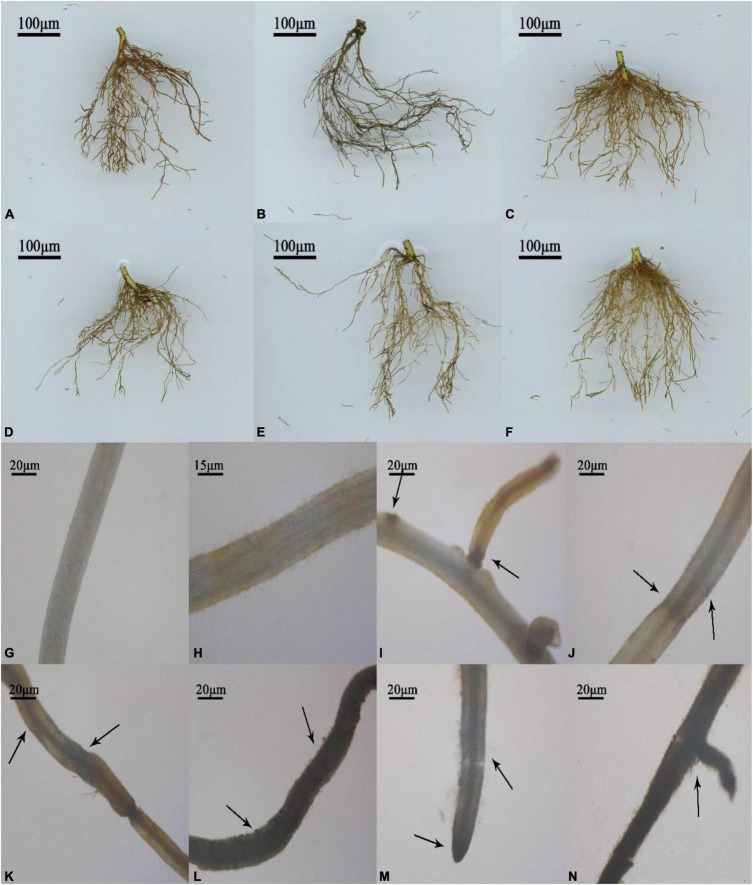
Root scans and pathology. **(A)** Infected T337; **(B)** Infected M26; **(C)** Infected 12-2; **(D)** Control T337; **(E)** Control M26; **(F)** Control 12-2; **(G)** The microscopic morphology of control PDB-treated 12-2 after 7 days. Control T337 and M26 were similar in appearance to control 12-2; **(H)** The microscopic morphology of 12-2 7 days after inoculation with *Fusarium proliferatum* MR5; **(I–K)** The microscopic morphology of T337 7 days after inoculation with *Fusarium proliferatum* MR5; **(L–N)** The microscopic morphology of M26 7 days after inoculation with *Fusarium proliferatum* MR5. A scale bar is shown in the figure.

As shown in Figure 2, the roots of T337, M26, and 12-2 tissue culture seedlings all showed varying degrees of damage 7 d after inoculation with MR5 spore solution. The root color changed to reddish brown (Figure 2A), dark gray (Figure 2B), or slightly red (Figure 2C), whereas the roots of tissue culture seedlings inoculated with PDB solution were bright yellow-brown (Figures 2D–F). The root systems of M26 showed the most severe damage, and some roots were even broken and rotted (Figures 2B,E). By contrast, the root systems of treated 12-2 plants showed no significant differences from those of control plants (Figures 2C,F). T337 showed an intermediate level of root system damage, and there were no signs of decay (Figures 2A,D). Under the stereomicroscope, the root systems inoculated with PDB solution appeared smooth, with no damaged spots and a brighter color (Figure 2G). After inoculation with MR5 spore solution, the roots of 12-2 appeared mildly injured with slight red spots (Figure 2H). The roots of T337 were darkened, the damaged portions shrank, and reddish-brown spots appeared (Figures 2J,K); the lateral roots were more severely damaged, displaying atrophy and necrosis (Figure 2I). M26 showed the most serious root damage: its roots became black with black spots and bulges on the surface (Figure 2L); the root system appeared broken, and hairy hyphae appeared (Figures 2M,N).

#### Root Architecture

Growth with MR5 spore solution did not affect the root architecture of T337, M26, or 12-2 (Table 9). MR5 had no effects on the root system length, surface area, volume, tip number, fork number, or diameter of T337, M26, and 12-2.

**TABLE 9:** Effects of *Fusarium proliferatum* MR5 on the root characteristics of 12-2, T337, and M26 apple rootstocks.

Treatment		Root length (mm)	Root area (mm^2^)	Root Volume (mm^3^)	Root tips number	Root forks number	Root diameter (mm/10)
T337	Infected	1483.96 ± 48.63	1877.42 ± 196.84	202.12 ± 17.67	250.33 ± 25.21	1093.00 ± 123.56	0.44 ± 0.02
	Control	1404.88 ± 59.87	1648.07 ± 67.53	183.79 ± 14.50	223.67 ± 9.02	972.33 ± 37.32	0.43 ± 0.01
M26	Infected	1325.48 ± 37.65	1606.91 ± 120.38	172.18 ± 24.40	210.33 ± 31.32	854.33 ± 73.99	0.38 ± 0.03
	Control	1320.16 ± 69.66	1611.64 ± 164.24	172.99 ± 25.69	331.67 ± 43.32	977.67 ± 28.32	0.38 ± 0.02
12-2	Infected	1520.71 ± 149.37	2122.73 ± 118.28	237.61 ± 14.92	347.00 ± 63.66	1111.67 ± 134.46	0.45 ± 0.03
	Control	1566.66 ± 66.24	2189.93 ± 198.57	245.25 ± 34.74	335.67 ± 32.74	1159.00 ± 73.82	0.44 ± 0.01

Within a rootstock, Student’s t-test was used to determine the significance of the difference between control and ARD soil. Data are expressed as mean ± SE. *p < 0.05; **p < 0.01.

#### Root Fresh and Dry Weights

Growth with MR5 spore solution did not affect the above- and belowground fresh and dry weights of T337, M26, and 12-2 (Table 10), and it had no significant effect on their root-to-shoot ratios.

**TABLE 10:** Effects of *Fusarium proliferatum* MR5 on the fresh and dry weights of 12-2, T337, and M26 apple rootstocks.

Treatment		Fresh weight (g)		Dry weight (g)		R/S
		Root	Branch	Root	Branch	
T337	Infected	0.26 ± 0.01	0.62 ± 0.01	0.05 ± 0.00	0.26 ± 0.01	0.20 ± 0.01
	Control	0.27 ± 0.01	0.65 ± 0.01	0.05 ± 0.00	0.26 ± 0.01	0.19 ± 0.01
M26	Infected	0.18 ± 0.00	0.54 ± 0.02	0.04 ± 0.00	0.16 ± 0.00	0.25 ± 0.01
	Control	0.18 ± 0.01	0.55 ± 0.01	0.04 ± 0.00	0.17 ± 0.01	0.24 ± 0.01
12-2	Infected	0.27 ± 0.00	0.78 ± 0.01	0.07 ± 0.00	0.30 ± 0.01	0.22 ± 0.02
	Control	0.27 ± 0.00	0.78 ± 0.02	0.06 ± 0.00	0.29 ± 0.02	0.22 ± 0.01

Within a rootstock, Student’s t-test was used to determine the significance of the difference between control and ARD soil. Data are expressed as mean ± SE. *p < 0.05; **p < 0.01. R/S indicates the root-to-shoot ratio. It is the value obtained by dividing the root dry weight by the shoot dry weight.

#### Root Metabolic Activity Measurements

**FIGURE 3:**
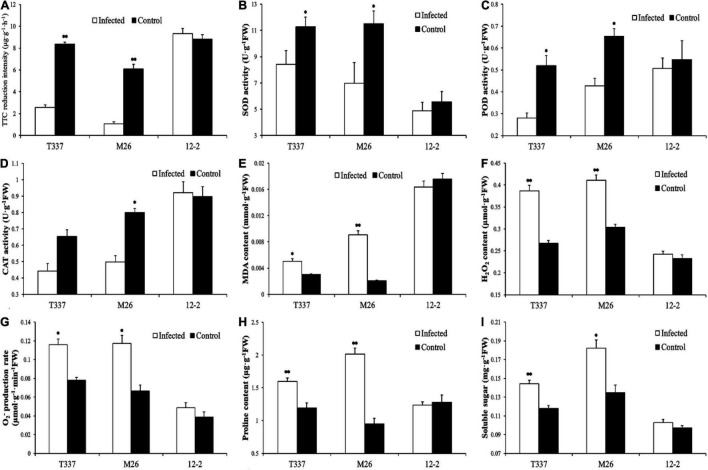
Effects of *Fusarium proliferatum* MR5 on multiple root parameters of 12-2, T337, and M26 apple rootstocks. **(A)** Root metabolic activity measurements; **(B–D)** Root antioxidant enzyme activity; **(E)** Root MDA content; **(F,G)** Root reactive oxygen species level; **(H,I)** Root permeable substance content. Within a rootstock, Student’s *t*-test was used to determine the significance of the difference between control and ARD soil. *p < 0.05; ^**^p < 0.01.

Growth with MR5 spore solution had a significant effect on the root metabolic activity of T337 and M26 (Figure 3A) but not on that of 12-2. Compared with their respective controls, the root metabolic activity of T337 and M26 decreased by 70.08 and 82.40%, respectively.

#### Root Antioxidant Enzyme Activity and Malondialdehyde Content

Growth with MR5 spore solution altered the antioxidant enzyme activity and MDA content of T337 and M26 roots (Figures 3B–E). MR5 significantly reduced SOD and POD activities in T337, significantly reduced the antioxidant enzyme activity of M26, and significantly increased the MDA contents of T337 and M26. MR5 had no significant effect on the antioxidant enzyme activity or MDA content of 12-2.

#### Root Reactive Oxygen Species Levels

Growth with MR5 spore solution increased H_2_O_2_ content and O_2_^–^ production rate of T337 and M26 roots (Figures 3F,G). Compared with controls, the H_2_O_2_ content and O_2_^–^ production rate of MR5-treated T337 roots increased by 44.84 and 48.82%, respectively, and those of M26 roots increased by 35.57 and 76.77%. MR5 had no significant effect on the reactive oxygen species levels of 12-2 roots.

#### Root Proline and Soluble Sugar Contents

Growth with MR5 spore solution significantly increased the proline and soluble sugar contents of T337 and M26 roots (Figures 3H,I). Compared with controls, the proline and soluble sugar contents of MR5-treated T337 roots increased by 33.76 and 22.43%, respectively, and those of M26 roots increased by 112.22 and 35.10%. MR5 had no significant effect on the root proline and soluble sugar contents of 12-2.

## Discussion

K^+^ can enhance the stress resistance of plants (Kenis and Keulemans, 2007), and the root system is the link between the plant and the soil. Meristem cells are sensitive, and the ion currents of the root system change rapidly and are closely related to plant stress resistance (Newman, 2001). Here, when the meristematic zones of T337, M26, and 12-2 roots were exposed to ARD soil extracts for 30 min, net K^+^ flux in the rhizoplane of M26 continued to decrease significantly, whereas 12-2 and T337 were able to recover K^+^ absorption to some extent, and the recovery of 12-2 was stronger than that of T337 (Supplementary Figure S1). It may be that under stress, 12-2 maintained intracellular K^+^ homeostasis by mobilizing K^+^ ions in the vacuole and other pools in root cells (Pinto and Ferreira, 2015; Zhang et al., 2020), activating the auxin pool in the endoplasmic reticulum to maintain normal root growth and development to deal with the damage caused by ARD (Friml and Jones, 2010). After 30 min of exposure, the ability of 12-2 to recover net K^+^ absorption in the elongation zone was also better than that of T337 and M26 (Supplementary Figure S2). 12-2 may therefore have maintained the turgor pressure and root cell expansion required by cells during root elongation by restoring the absorption of K^+^ (Dolan and Davies, 2004; Hu et al., 2020), thereby promoting root elongation, increasing root volume, and quickly responding to stress (Li et al., 2011). In the root mature zone of 12-2, net K^+^ influx increased significantly after exposure to ARD extract for 5 min, but it decreased significantly after 30 min, consistent with the study of Wang H. Y. et al. (2018) (Supplementary Figure S3). After 5 min of exposure, ARD may have induced a rapid increase in H_2_O_2_ in 12-2 root cells, and various root K^+^ osmotic channels are known to be activated by H_2_O_2_ (Wang H. Y. et al., 2018). H_2_O_2_ produced in roots can interact with transition metals (Fe^2+^ or Cu^2+^) to produce highly reactive OH⋅, thereby activating various K^+^ permeation channels and causing large amounts of K^+^ efflux (Shabala and Pottosin, 2014). After 30 min of exposure, the rapid accumulation of H_2_O_2_ in the mature zone of 12-2 roots may induce plant resistance. The subsequent removal of H_2_O_2_ may have induced the closure of K^+^ permeation channels, thereby reducing K^+^ outflow (Pinto and Ferreira, 2015; Wang H. Y. et al., 2018). On the other hand, the K^+^ accumulated in the root maturation zone may be transported to other root cells over time (Zhang et al., 2020), maintaining K^+^ homeostasis and thereby enhancing stress resistance (Pinto and Ferreira, 2015; Assaha et al., 2017). However, ARD affected K^+^ absorption of T337 and M26 mature root zones, causing a decrease in net K^+^ influx (Liu et al., 2012). Therefore, it appears that the 12-2 root system can quickly adjust its K^+^ homeostasis, maintain a steady state, and enhance ARD resistance.

Ca^2+^ plays an important role in plant stress signal transduction (Xu et al., 2018). In this experiment, ARD-30 caused a significant increase in the net efflux of Ca^2+^ from the root meristems of T337 and M26, whereas the net efflux of Ca^2+^ was basically unchanged in 12-2 (Supplementary Figure S4). Substantial Ca^2+^ efflux is not conducive to calcium signal transduction and affects the normal plant stress response (Feng et al., 2016). The relative homeostasis of Ca^2+^ in 12-2 roots may allow for rapid activation or inhibition of various membrane ion channels or specific enzymes, induce the expression of antiretroviral genes, stabilize cell walls and cell membranes, and thereby enhance environmental adaptation and improve ARD resistance (Roelfsema and Hedrich, 2010; Qi and Zhang, 2020). Five minutes of exposure to ARD soil extract had little effect on net efflux of Ca^2+^ in the rhizoplane of the three rootstocks. This may be because short-term ARD-induced Ca^2+^ efflux is mediated by Ca^2+^ channels (Kuster et al., 2011), and the ARD-5 treatment did not reach the specific threshold of Ca^2+^ stress that stimulates the meristem (Bose et al., 2011). With increasing exposure time of the root elongation zone, net Ca^2+^ efflux from 12-2 first increased and then decreased, in contrast to the continuously increasing net efflux from M26 and T337 (Supplementary Figure S5). This result suggested that ARD soil extract may weaken the growth of T337 and M26 roots in response to gravity, thereby destroying the steady state of Ca^2+^. Because 12-2 was less affected, it could maintain root cap gravity perception and the resulting asymmetric growth that occurs during root elongation, thereby quickly restoring steady-state Ca^2+^ concentration (Sobol and Kordyum, 2009; Dodd et al., 2010). These results were consistent with the root physiology and structure measurements, although the specific underlying mechanisms remain to be explored. The change in Ca^2+^ flux in the mature root zone of 12-2 was opposite to that of T337 and M26 (Supplementary Figure S6). ARD may therefore have affected the Ca^2+^/H^+^ exchanger and Ca^2+^-ATPase of T337 and M26. Ca^2+^ release into the cell or into intracellular organelles may have been inhibited, maintaining cellular Ca^2+^ concentrations at a higher level (Bose et al., 2011). Higher concentrations of Ca^2+^ could then trigger the aggregation of proteins and nucleic acids, resulting in the precipitation of phosphate, which is not conducive to plant health (Case et al., 2007). The mature zones of 12-2 may have had an efficient transport system that reduced Ca^2+^ efflux and maintained the steady state of the microenvironment (Feng et al., 2016). Therefore, we speculate that 12-2 may be able to quickly exchange information with other tissues and organs under ARD, maintaining the stability of Ca^2+^ at a specific time and location and thereby enhancing ARD tolerance.

Changes in root physiological indices in the pot experiment and the *Fusarium* tolerance test also showed that 12-2 had greater tolerance to ARD than the other tested rootstocks. Compared with its untreated control, 12-2 maintained high root growth and a large root surface area in replanted soil, and its root physiological indices differed little from those observed in sterilized soil. This suggested that 12-2 could more quickly perceive stress signals and regulate the expression of stress-related genes through signal transduction pathways, thereby regulating its physiological state and the distribution of metabolites among organs (Lager et al., 2010; Ghonaim et al., 2021) and maintaining the total length and surface area of the root system to cope with stress (Comas et al., 2013; Chen et al., 2020). In addition, maintenance of a high root growth rate in 12-2 may have reduced ARD sensitivity, limited infection by harmful fungi (Emmett et al., 2014), maintained root structure and function (Atucha et al., 2014), reduced root rot and browning (Ghosh and Xu, 2014), and promoted normal growth and increased ARD resistance. Under ARD stress, antioxidant enzyme activities were significantly lower in T337 and M26 roots from replanted soil than in those from sterilized soil, and high levels of MDA accumulated. By contrast, there were no significant differences in antioxidant enzyme activity and MDA content between the two treatments for 12-2 (Figure 1). It may be that under ARD, superoxide (O_2_^⋅^) and other harmful substances accumulate in larger amounts in M26 and T337 (Zhang et al., 2013; Berni et al., 2019), exceeding their cellular tolerance (Zhang et al., 2021). Although SOD, POD, and CAT work together to disproportionate O_2_^⋅^ to H_2_O_2_ and O_2_, reducing its toxicity to a certain extent (Sharma et al., 2012; Li et al., 2019), the H_2_O_2_ produced by disproportionation will accumulate to excessively high levels over time, leading to massive production of the membrane lipid peroxidation product MDA (Gill and Tuteja, 2010). MDA reacts with and denatures cell macromolecules such as proteins and nucleic acids, thereby destroying cell structure and function (Zhao et al., 2019), rapidly inhibiting cell antioxidant enzyme activity, and ultimately leading to cell death (Ayala et al., 2014; Mu et al., 2021). Therefore, within one week of infection by ARD-associated *F. proliferatum* MR5, the roots of M26 and T337 showed numerous brown spots, rot, and fractures (Figure 2). These observations were consistent with previous reports of epidermal cell lysis, disintegration of cortical cells, root tip necrosis, and the almost complete loss of functional root hairs due to excessive accumulation of reactive oxygen species (Petrov et al., 2015; Han et al., 2021). Unlike the other two rootstocks, 12-2 could maintain a degree of balance between ROS production and scavenging, thereby maintaining the normal cellular redox state (Tanveer and Shah, 2017). ROS induce the accumulation of large amounts of lignin and callosin near the host infection site, participate in the oxidative cross-linking of cell wall structural proteins, and produce insoluble dimers and tetramers that are deposited on the cell wall, thereby strengthening it and enhancing the plant’s mechanical barrier against pathogens (Sharma et al., 2012; Castro et al., 2021). The significant increase in soluble sugars and proline were also consistent with the poor ARD tolerance of M26 and T337 roots, which led to excessive production of ROS and excessive oxidative damage to lipid membranes (Sharma et al., 2012; Yang et al., 2015); lack of accumulation of these stress-related metabolites in 12-2 provided further evidence that it had improved ARD tolerance.

The results presented here suggest that 12-2 has a certain degree of ARD resistance and tolerance to ARD-associated *F. proliferatum* MR5; nonetheless, the molecular mechanisms by which 12-2 responds to ARD and MR5 are still unknown. *Fusarium* is the main harmful fungus that causes ARD in China, but it is unclear whether 12-2 is also resistant to oomycetes such as *Pythium* and *Phytophthora*. To further assess the performance of 12-2, it will be necessary to perform further comparisons with other elite rootstocks. Many experiments must be performed on new rootstocks, including evaluations of their graft compatibility, fruit yield, and survival and growth in other planting regions. These topics will be the focus of future research.

## Conclusion

Measurements of K^+^ and Ca^2+^ ion currents in the rhizoplane meristem, elongation, and maturation zones suggested that 12-2 exhibited good resistance to ARD in each area of the root. And there were no significant differences in the root physiological parameters of 12-2 rootstock in replanted or sterilized soil. A pathogen infection test also showed that 12-2 had good resistance to ARD-associated *F. proliferatum* MR5. ARD had a greater impact on K^+^ and Ca^2+^ ion currents in various root zones of T337 and M26 and on most root physiological parameters. T337 and M26 showed a degree of intolerance to ARD-associated *F. proliferatum* MR5. 12-2 could be used as an important material for the breeding of ARD-resistant apple rootstocks, which will be important for fundamentally solving the problem of ARD in China.

## Data Availability Statement

The original contributions presented in the study are included in the article/Supplementary Material, further inquiries can be directed to the corresponding author.

## Author Contributions

YM and XSh planned and designed the research. YM, YY, XC, HW, XSu, XQ, and YL performed experiments, conducted fieldwork, and analyzed data. YM, YY, YH, and XSh wrote the manuscript. All authors contributed to the article and approved the submitted version.

## Author Disclaimer

All claims expressed in this article are solely those of the authors and do not necessarily represent those of their affiliated organizations, or those of the publisher, the editors and the reviewers. Any product that may be evaluated in this article, or claim that may be made by its manufacturer, is not guaranteed or endorsed by the publisher.

## Conflict of Interest

The authors declare that the research was conducted in the absence of any commercial or financial relationships that could be construed as a potential conflict of interest.

## Publisher’s Note

All claims expressed in this article are solely those of the authors and do not necessarily represent those of their affiliated organizations, or those of the publisher, the editors and the reviewers. Any product that may be evaluated in this article, or claim that may be made by its manufacturer, is not guaranteed or endorsed by the publisher.

## Abbreviations

ARD: apple replant disease
12-2: elite apple rootstock line 12-2
MR5: *F. proliferatum* MR5
SOD: superoxide dismutase
POD: peroxidase
CAT: catalase
MDA: malondialdehyde
R/S: root-to-shoot ratio
Before-ARD: net rhizoplane ion flow before ARD
ARD-5: net rhizoplane ion flow after immersion in ARD soil extract for 5 min
ARD-30: net rhizoplane ion flow after immersion in ARD soil extract for 30 min.
