# Supplementary material for: Detection of Root Physiological Parameters and Potassium and Calcium Currents in the Rhizoplane of the Apple Rootstock Superior Line 12-2 With Improved Apple Replant Disease Resistance
Source: Front Plant Sci. 2021 Dec 17;12:734430. doi: 10.3389/fpls.2021.734430 (PMC8718911; doi:10.3389/fpls.2021.734430)
Supplement: Supplementary file 1 [file Data_Sheet_1.ZIP › data sheet 1/Supplmentary Material/Supplementary Material .doc]

# Supplementary Material

**Figure S1.** Effects of ARD on K^+^ uptake in the root meristem zone (400 µm from the root tip). (a): T337 net K^+^ flux in meristem zone; (b): M26 net K^+^ flux in meristem zone; (c): 12-2 net K^+^ flux in meristem zone; (d): Effects of ARD on net K^+^ flux of three rootstocks in meristem zone.

Note: Before-ARD: net rhizoplane ion flow before ARD; ARD-5: net rhizoplane ion flow after immersion in ARD soil extract for 5 min; ARD-30: net rhizoplane ion flow after immersion in ARD soil extract for 30 min. Different letters in the column show significant differences (P < 0.05). The same conventions are used in Figures 2–3, below.

**Figure S2.** Effects of ARD on K^+^ uptake in the root elongation zone (1000 µm from the tip). (a): T337 net K^+^ flux in elongation zone; (b): M26 net K^+^ flux in elongation zone; (c): 12-2 net K^+^ flux in elongation zone; (d): Effects of ARD on net K^+^ flux of three rootstocks in elongation zone.

**Figure S3.** Effects of ARD on K^+^ uptake in the root mature zone (4200 µm from the tip). (a): T337 net K^+^ flux in mature zone; (b): M26 net K^+^ flux in mature zone; (c): 12-2 net K^+^ flux in mature zone; (d): Effects of ARD on net K^+^ flux of three rootstocks in mature zone.
